# The capacity to consent to treatment is altered in suicidal patients

**DOI:** 10.1186/s12991-023-00459-w

**Published:** 2023-09-09

**Authors:** Emilie Olié, Thomas Catanzaro, Manon Malestroit, Julio A. Guija, Lucas Giner, Philippe Courtet

**Affiliations:** 1 Department of Emergency Psychiatry and Acute Care, Lapeyronie Hospital, CHU Montpellier, Montpellier, France; 2 IGF, Univ. Montpellier, CNRS, INSERM, Montpellier, France; 3 Department of Psychiatry, Universidad de Sevilla, Seville, Spain; 4 Service of Psychiatry. Institute of Legal Medicine, Seville, Spain

**Keywords:** Capacity to consent, Decision-making capacity, MacCAT-T, Depression, Suicide attempt

## Abstract

**Background:**

Many patients with depression refuse treatment. Moreover, suicide attempters often display low perceived need of treatment and impaired decision-making. These observations raise questions about the capacity to treatment consent in depressed suicide attempters (SA).

**Methods:**

In patients with current depressive episode (*N* = 33 SAs and *N* = 27 non-SAs), consent capacity was evaluated with the MacArthur Competence Assessment Tool for Treatment (MacCAT-T), insight with the Beck Cognitive Insight Scale, and depression severity with the Beck Depression Inventory (BDI).

**Results:**

The median BDI score in the whole sample (*N* = 60) was 21 [10;36], and was higher in SAs than non-SAs (27 [11;36] vs. 15 [10:33], *p* < 0.001). Consent capacity was impaired in 30% (appreciation), 53% (reasoning) and 60% (understanding) of all patients. MacCAT-T sub-scores were lower in SAs than non-SAs (understanding: 4.4 [2.35;5.8] vs. 5.3 [3.13;6]); appreciation: 3 [1;4] vs. 4 [2;4]); reasoning (4 [1;7] vs. 7 [3;8]), and ability to express a choice: 1 [0;2] vs. 2 [0;2]; all *p* < 0.001). In multivariate analyses, suicide attempt history and depression severity (but not insight) were negatively associated with MacCAT-T sub-scores.

**Conclusion:**

More research is needed on the capacity to consent to treatment of patients with depression, particularly suicidal individuals, to make informed choices about their treatment.

*Trial registration* The Montpellier University Hospital Institutional Review Board approved the study (No. 202100714).

## Introduction

Depression is diagnosed in ~ 6% of the adult population worldwide each year and is one of the top ten causes of disability-adjusted life years (DALYs) between 10- and 49-year-old individuals [1]. Depression is also one of the leading causes of suicide worldwide featured by a causal link but also neurobiological dysfunctions, such as immune-inflammatory changes [2] or common risk factors such as cannabis use [3]. The management of depression is thus a mental health priority. However, between 35.5% and 50.3% of patients with severe depression do not receive any treatment [4]. This suggests that some patients may refuse all medical interventions despite their clear need for help [5]. Appelbaum and Roth [4] wrote that *“Of all the psychopathological processes associated with refusal [of treatment], depression is the most difficult to recognize, because it masquerades as ‘Just the way I would think if it happened to me’ … The depressed patient is frequently able to offer ‘rational’ explanations for the choices that are made.”* This raises questions concerning the treatment decision-making capacity (DMC) of patients with depression. In a princeps study, Grisso and Appelbaum (1995) reported that DMC was impaired in 25% of depressed participants. They also developed the MacArthur Competence Assessment Tool for Treatment (MacCAT-T [7]) to assess DMC based on four competence categories: understanding, appreciating, reasoning, and expressing a choice. This semi-structured tool is used to assess the degree to which patients understand the information and recognize (appreciate) the relevance of the information on their situation, their reasoning capacity, and whether they can express a choice. If one of these four areas is impaired, a patient may be considered unable to exercise their autonomy relative to treatment decision-making. Previous studies used the MacCAT-T in patients with depression, with heterogeneous results, as highlighted in the systematic review by Hindmarch et al. [8]. Some studies concluded that in patients with depression, capacity measures are not impaired [9, 10], whereas others found impairment in up to 30% of participants [11, 12]. Appreciation, which requires comparing several alternatives, seems to be the most altered DMC component in individuals with depression. Moreover, some depression characteristics, such as the episode severity [11] and psychotic features [13], may influence DMC components.

The suicide attempt history has never been considered in studies on the capacity to consent to treatment, although it is associated with depression severity [14] and cognitive impairment that may affect DMC. Indeed, suicide attempters display impaired decision-making abilities [15], lower problem-solving leading to feelings of entrapment [16, 17], and lack of future positive thinking [18].

Therefore, the aim of this study was to compare DMC using the MacCAT-T in patients with current depressive episode and with/without history of suicide attempt. We hypothesize that (1) suicide attempters are more likely than non-attempters to have DMC alterations independently of the severity of depressive symptomatology; (2) an association between poor insight and low DMC in depressed patients.

## Materials and methods

### Study population

The sample included 60 patients (age 18 to 75 years) with current major depressive episode, according to the Diagnostic and Statistical Manual of Mental Disorders, fifth edition [19], and with a Beck Depression Inventory (BDI) score > 9, recruited at Le Mas Careiron Public Psychiatric Hospital, Uzes (France), and at the Department of Psychiatric Emergency and Acute Care, University Hospital of Montpellier (France) from the 1st February to 31st July 2021. Patients with lifetime history of schizophrenia or schizoaffective disorder, or current psychotic features were not included.

### Clinical assessment

A trained psychiatrist (T.C.) assessed current psychopathology using the Mini International Neuropsychiatric Interview (MINI-7) [20], lifetime history of suicide attempt, and DMC using the MacCAT-T [21]. The MacCAT-T is a validated semi-structured interview to assess DMC providing relevant information disclosures to patients about their illness, the nature of treatment options, and their risks and benefits. Assessment of the MacCAT-T consists of four summary scores, which are named according to the dimensions of the treatment-related decision-making capacity as follows: (1) understanding (the ability to understand disclosed information); (2) appreciation (the ability to appreciate the significance of the disclosed information for one’s own condition and situation); (3) reasoning (about the potential risks and benefits of one’s choices, e.g., in comparing the risks and benefits of treatment options); and (4) expression of a choice (the ability to communicate a choice regarding a proposed treatment). Each summary score has a different score range: 0–6 for understanding, 0–4 for appreciation, 0–8 for reasoning. Intraclass correlations for the MacCAT-T sub-scores were 0.99 for understanding, 0.87 for appreciation, and 0.91 for reasoning. Higher sub-scores indicate greater decisional capacity. Adequate decisional capacity was operationalized according to the sub-score cut-offs [22, 23], in line with previous research [7]: ≥ 5 for understanding, ≥ 3 for appreciation, and ≥ 6 for reasoning.

Patients completed the self-report BDI [24] to assess depressive symptomatology, and the Beck Cognitive Insight Scale (BCIS) to evaluate insight [25]. As previously described [25], insight was quantified using the BCIS composite index that is calculated by subtracting the self-certainty subscale score from the self-reflectiveness subscale score. Sociodemographic data, number of past depressive episodes, and current psychotropic treatments (drug, dose) also were recorded.

The study was conducted in accordance with the CONSORT ethical guidelines. Informed written consent was obtained from all participants. The Montpellier University Hospital Institutional Review Board approved the study (No. 202100714). The trial was registered as NCT03052855 in clinicaltrial.gov.

### Statistical analyses

Descriptive statistics were used to characterize the sample. Continuous variables were compared between patients with and without lifetime history of suicide attempt using the Mann–Whitney’s *U* test. Categorical data were compared with the Chi-square or Fisher’s exact probability tests and the alpha level was set at 0.05.

Multivariate regression models were used to determine the association of depression, insight, and suicide attempt history with each MacCAT-T subscale as the dependent variable and as continuous variable due to the sample size.

## Results

### Sample characteristics

The sample characteristics are described in Table 1. The median BDI score was 21 (min: 10–max: 36). The median MacCAT-T sub-scores were: 4.62 [2.35;6] for understanding, 3 [1;4] for appreciation, 5.5 [1;8] for reasoning, and 2 [0;2] for expressing a choice. Understanding was impaired in 36 (60%) patients, appreciation in 18 (30%) patients, n 32 (53%) patients. The median BCIS composite index was 2 [− 15;22].

**Table 1:** Description of the sample

Variables	Whole sample	No suicide attempt	Suicide attempt	*P* value
	*N* = 60	*N* = 27	*N* = 33	
	Median [Min;Max]	Median [Min;Max]	Median [Min;Max]	
	*N* (%)	*N* (%)	*N* (%)	
Age	42 [18;74]	42 [19;73]	38 [18;74]	0.853
Women	33 (55%)	15 (55.6%)	18 (54.5%)	> 0.999
Psychopathology				
Number of depressive episodes	2 [1;5]	1 [1;4]	2 [1;5]	0.006
Current anxiety disorder	20 (33%)	12 (44.4%)	8 (24.2%)	0.169
Bipolar disorder	8 (13%)	1 (3.7%)	7 (21.2%)	0.063
Alcohol use disorder/dependence	6 (10%)	2 (7.4%)	4 (12.1%)	0.681
Substance use disorder/dependence	8 (13%)	3 (11.1%)	5 (15.2%)	0.719
BDI total score	21 [10;36]	15 [10:33]	27 [11;36]	< 0.001
BCIS composite index	2 [− 15;22]	6 [− 12;22]	− 3 [− 15;19]	0.042
MacCAT-T sub-scores				
Understanding	4.6 [2.35;6]	5.3 [3.13;6]	4.4 [2.35;5.8]	0.001
Appreciation	3 [1;4]	4 [2;4]	3 [1;4]	< 0.001
Reasoning	5 [1;8]	7 [3;8]	4 [1;7]	< 0.001
Expressing a choice	1 [0;2]	2 [0;2]	1 [0;2]	< 0.001
Pharmacological treatments				
Hypnotics	6 (10%)	3 (11.1%)	3 (9.1%)	> 0.999
Anxiolytics	39 (65%)	13 (48.1%)	26 (78.8%)	0.028
Antidepressants	55 (92%)	25 (92.6%)	30 (90.9%)	> 0.999
Anticonvulsants	4 (7%)	1 (3.7%)	3 (9.1%)	0.620
Antipsychotic	23 (38%)	9 (33.3%)	14 (42.4%)	0.650
Lithium salts	5 (8%)	2 (7.4%)	3 (9.1%)	> 0.999

*BCIS*, Beck Cognitive Insight Scale; *BDI*, Beck Depression Scale

### Impact of history of suicide attempt on DMC

#### Univariate analyses

Thirty-three (55%) patients had lifetime history of suicide attempts (Table 1). Compared with patients without suicide attempt history, number of past depressive episodes (1 [1;4] vs 2 1;5], *p* = 0.006) and depression severity (15 [10;23] vs 27 [11;36] *p* < 0.001) were higher, whereas the BCIS composite index (6 [− 12;22] vs − 3 [− 15;19], *p* = 0.042) was lower in patients with suicide attempt history. Moreover, the MacCAT-T sub-scores were lower in patients with than without suicide attempt history: Understanding (5.3 [3;13.6] vs 4.4 [2.35;8]; *p* = 0.001), Appreciation: (4 [2;4] vs. 3 [1;4]; *p* < 0.001), Reasoning: (7 [3;8] vs 4 [1;7]; *p* < 0.001), and Expressing a choice: (2 [0;2] vs 1 [0;2]; *p* < 0.001).

#### Multivariate analyses

Depression severity, but not insight, was negatively associated with all Mac-CAT-T sub-scores (Table 2). History of suicide attempt was negatively associated with the following MacCAT-T sub-scores: appreciation (beta (95% CI) = − 0.68 [− 1.0;− 0.34], *p* < 0.001), reasoning (beta (95% CI) = − 1.1 [− 1.8;− 0.33], *p* = 0.007), and expressing a choice (beta (95% CI) = − 0.52 [− 0.79;− 0.24], *p* = 0.001).

**Table 2:** Associations between MacCAT-T sub-scores, history of suicide attempt, depression and insight (multivariate analysis)

	MacCAT-T understanding			MacCAT-T appreciation			MacCAT-T reasoning			MacCAT-T choice		
	Beta	95% CI	*p*-value	Beta	95% CI	*p*-value	Beta	95% CI	*p*-value	Beta	95% CI	*p*-value
BDI score	− 0.05	− 0.08, − 0.01	0.009	− 0.04	− 0.06, − 0.01	0.003	− 0.13	− 0.18, − 0.08	< 0.001	− 0.04	− 0.06, − 0.02	< 0.001
BCIS index	0.00	− 0.02, 0.03	0.8	0.01	− 0.01, 0.03	0.3	0.02	− 0.02, 0.06	0.4	0.01	− 0.01, 0.02	0.4
Past SA	− 0.45	− 0.94, 0.04	0.076	− 0.68	− 1.0, − 0.34	< 0.001	− 1.1	− 1.8, − 0.33	0.007	− 0.52	− 0.79, − 0.24	< 0.001

*BCIS*, Beck Cognitive Insight Scale composite index; *BDI*, Beck Depression Scale total score; *CI*, Confidence Interval; *SA*, suicide attempt

## Discussion

Our results strengthen the hypothesis of an impaired DMC in some patients with depression [6], associated with higher depressive symptomatology. DMC (at least one MacCAT-T sub-score) was impaired in more than 30% of patients. The mean MacCAT-T sub-scores were close to the previously reported scores in patients with depression [23] and in patients with anorexia nervosa [26], but higher than those of patients with schizophrenia [27]. Our results add that history of suicide attempt is associated to impaired DMC in depressed patients. Although more research is needed, assessing the capacity to consent in individuals with depression should become a crucial component of their routine clinical evaluation. Decisions made by these patients could negatively affect their adherence to treatment and ability to accept adequate treatment. Moreover, it is known that suicidal patients are less likely to seek help [28, 29], frequently because of low perceived need of treatment and perceived inefficiency of treatments [28]. Identifying DMC alterations is crucial to determine whether treatment refusal should be respected by health professionals [30]. Our results also raise the issue of access to euthanasia or assisted suicide (EAS) for these patients. Based on Dutch case reports of patients who received EAS for psychiatric conditions, the capacity to consent (only focused on the global judgment of the patients’ capacity) was assessed in 55% of patients [31], although most patients were depressed and with history of suicide attempts [32]. Importantly, many patients had refused potential effective treatments before requesting and receiving EAS. In this case, In this case, using the MacCAT-T to adequately assess the capacity to consent in those patients could have prevented EAS. In this case, using the MacCAT-T to adequately assess the capacity to consent in those patients could have prevented EAS.

Our study did not find any association between insight and DMC. In our sample, the positive correlation between insight level and depression severity (results not shown) may explain this negative finding. However, this negative result is in accordance with Owen et al. who reported that: (1) insight mapped poorly to DMC in depression (but it mapped well in schizophrenia) [33]; and (2) insight gain may not be a good indicator of regaining capacity to consent in patients with depression [34].

Our study has limitations. First, our sample was small, but it was sufficient to detect significant associations between depression severity, suicide attempt history (but not insight), and poor DMC. Second, using the BCIS to measure insight may be debatable because this tool was validated in patients with psychosis and may not be adequate for patients with depression [25]. Third, only inpatients were included. This limits the generalization of our results and may explain the high rate of DMC impairment (up to 60%). Fourth, cognitive measures, such as mental flexibility and decision-making under uncertainty (assessed with the Iowa Gambling Task) that could potentially affect the capacity to consent, also should have been taken into account.

## Conclusion

In their daily practice, clinicians should be aware that the assessment of the capacity to consent is a challenging task which deserves a rigorous investigation. This may rely on standardized tools such as Mac-CAT-T, whose score does not depend on the severity of depressive symptomatology or insight. Such assessment may help to better balance benefit/risk ratio of treatments, to improve educational messages and to prevent negative consequences of refusals related to altered mental capacity. More research is needed on the DMC of patients with depression, especially suicidal ones, to identify those at higher to refuse treatments and to develop specific strategies to improve acceptance.

## Data Availability

The data and study material are available on request of the corresponding author (e-olie@chu-montpellier.fr).
